# Medial prefrontal cortex suppresses reward-seeking behavior with risk of punishment by reducing sensitivity to reward

**DOI:** 10.3389/fnins.2024.1412509

**Published:** 2024-06-05

**Authors:** Monami Nishio, Masashi Kondo, Eriko Yoshida, Masanori Matsuzaki

**Affiliations:** ^1^Department of Physiology, Graduate School of Medicine, The University of Tokyo, Tokyo, Japan; ^2^Department of Biological Sciences, Graduate School of Science, The University of Tokyo, Tokyo, Japan; ^3^International Research Center for Neurointelligence (WPI-IRCN), The University of Tokyo Institutes for Advanced Study, Tokyo, Japan; ^4^Brain Functional Dynamics Collaboration Laboratory, RIKEN Center for Brain Science, Saitama, Japan

**Keywords:** punishment, reward omission, medial prefrontal cortex, reinforcement learning, pharmacological inhibition

## Abstract

Reward-seeking behavior is frequently associated with risk of punishment. There are two types of punishment: positive punishment, which is defined as addition of an aversive stimulus, and negative punishment, involves the omission of a rewarding outcome. Although the medial prefrontal cortex (mPFC) is important in avoiding punishment, whether it is important for avoiding both positive and negative punishment and how it contributes to such avoidance are not clear. In this study, we trained male mice to perform decision-making tasks under the risks of positive (air-puff stimulus) and negative (reward omission) punishment, and modeled their behavior with reinforcement learning. Following the training, we pharmacologically inhibited the mPFC. We found that pharmacological inactivation of mPFC enhanced the reward-seeking choice under the risk of positive, but not negative, punishment. In reinforcement learning models, this behavioral change was well-explained as an increase in sensitivity to reward, rather than a decrease in the strength of aversion to punishment. Our results suggest that mPFC suppresses reward-seeking behavior by reducing sensitivity to reward under the risk of positive punishment.

## 1 Introduction

Consider decision making in the following situation: you know there may be candy in the kitchen. But if you go to the kitchen, it may not be there. Or, you may find it and eat it and get scolded later by your mother. Now what do you do? Actions motivated by rewards are frequently associated with the risk of punishment (Park and Moghaddam, 2017). In this context, a punishment is described as an adverse consequence resulting from a reward-seeking behavior, and it can profoundly affect subsequent behavior. Punishment can be thought of as being either positive or negative, with positive punishment in which an unpleasant outcome (e.g., a stimulus that causes discomfort or pain) is added and negative punishment in which a reinforcing consequence is omitted (e.g., increase in risk of loss or reinforcer uncertainty) (Palminteri and Pessiglione, 2017; Jean-Richard-Dit-Bressel et al., 2018; Piantadosi et al., 2021). Both of them reduce the probability that the same behavior will be produced again and increase the exploration of less risky alternatives. However, in a previous study (Tanimoto et al., 2020), we demonstrated that the impact of negative punishment (reward omission) on mice behavior is better explained as a covert reward, a reinforcer of “doing nothing,” rather than a punishment, a negative reinforcer of “doing,” in the context of reinforcement learning. This finding suggests the potential for distinct coding of positive and negative punishment in our brain.

It is well known that the medial prefrontal cortex (mPFC) is involved in decision-making during approach-avoidance conflicts (Bechara et al., 2002; Xue et al., 2009; Chen et al., 2013; Friedman et al., 2015; Kim et al., 2017; Monosov, 2017; Siciliano et al., 2019; Fernandez-Leon et al., 2021; Bloem et al., 2022; Jacobs et al., 2022). When the dorsomedial prefrontal cortex (dmPFC) strongly responds to risk, humans prefer less risky choices (Xue et al., 2009), and patients with a ventromedial prefrontal cortex (vmPFC) lesion can show hypersensitivity to reward (Bechara et al., 2002). Many neurons in the macaque anterior cingulate cortex (ACC) represent the value and uncertainty of rewards and punishments (Monosov, 2017). In rats, the silencing of mPFC neurons promotes cocaine-seeking behavior under the risk of foot shock (Chen et al., 2013), and binge drinking of alcohol under the risk of a bitter tastant (quinine) (Siciliano et al., 2019), whereas activating the mPFC can attenuate these behaviors. mPFC neurons that project to the nucleus accumbens are suppressed prior to reward-seeking behavior with foot-shock punishment, while their activation reduces reward seeking (Kim et al., 2009). Thus, mPFC is obviously involved in decision-making under a risk of positive punishment.

There are also a few studies that have examined the functions of mPFC under risk of negative punishment. For example, mPFC is known to be required for updating the choice bias in a choice task between a small reward with certainty and a large reward with high omission probability (St. Onge and Floresco, 2010; Piantadosi et al., 2021). However, it has not been examined whether mPFC is equally involved in decision-making with positive and negative punishments in very similar tasks. In addition, it remains unclear which variable in the model that could explain the behavior of avoiding positive or negative punishment is related to mPFC.

In this study, we constructed a lever-pull task in head-fixed male mice with either a positive punishment (air-puff stimulus) or a negative punishment (reward omission) and utilized a reinforcement learning model to identify the variables influencing behavior in each condition. Following training, we investigated the impact of pharmacological inactivation of mPFC on the lever pull probability. Finally, we modeled their behavior under mPFC inhibition by adjusting reinforcement learning parameters, aiming to elucidate the role of the mPFC in each condition.

In summary, this study utilized a reinforcement learning model to uncover a unique coding mechanism for different types of punishment and to elucidate the role of the mPFC in these processes. We found that the effect of positive and negative punishment on behavior are modeled differently with reinforcement learning model. Moreover, we found mPFC is necessary for behavioral inhibition under the risk of positive punishment, but not negative punishment. Additionally, we introduced an analytical method to explain the behavioral effect of mPFC inactivation in the form of a change of parameters in the context of reinforcement learning. Our analyses suggested that the effect of mPFC inactivation was best explained as an increase in sensitivity to reward, rather than as a decrease in the strength of the aversion to positive punishment or a decrease in the covert reward to non-action.

## 2 Materials and methods

### 2.1 Animals

All animal experiments were approved by the Animal Experimental Committee of the University of Tokyo. C57BL/6 mice (male, aged 2–3 months at the start of behavioral training, SLC, Shizuoka, Japan, RRID: MGI:5488963) were used for the experiments. These mice had not been used in other experiments before this study. All mice were provided with food and water *ad libitum* and housed in a 12:12 h light–dark cycle (light cycle; 8 a.m.–8 p.m.). All behavioral sessions were conducted during the light period.

### 2.2 Head plate implantation

Mice were anesthetized by intramuscular injection of ketamine (74 mg/kg) and xylazine (10 mg/kg), with atropine (0.5 mg/kg) injected to reduce bronchial secretion and improve breathing, an eye ointment (Tarivid; 0.3% w/v ofloxacin; Santen Pharmaceutical, Osaka, Japan) applied to prevent eye-drying, and lidocaine jelly applied to the scalp to reduce pain. Body temperature was maintained at 36°C–37°C with a heating pad. After the exposed skull was cleaned, an incision was made in the skin covering the neocortex, and a custom head plate (Tsukasa Giken, Shizuoka, Japan) was attached to the skull using dental cement (Fuji lute BC; GC, Tokyo, Japan; and Estecem II; Tokuyama Dental, Tokyo, Japan). The surface of the intact skull was coated with dental adhesive resin cement (Super bond; Sun Medical, Shiga, Japan) to prevent drying. A single intraperitoneal injection of the anti-inflammatory analgesic carprofen (5 mg/kg, Rimadile; Zoetis, NJ, United States) was given after all surgical procedures. Mice were allowed to recover for 3–5 days before behavioral training.

### 2.3 Behavioral training

After recovery from the head plate implantation, the mice were water-deprived in their home cages. Each mouse received about 1 mL of water per session per day, but they were sometimes given additional water to maintain their body weight at 85% of their initial weight throughout the experiments. The mice were usually trained for five consecutive days per week and were given 1 mL of water on days without training. The behavioral apparatus (sound attenuation chamber, head-fixing frame, body holder, sound presentation system, water-supply system, and integrated lever device) was manufactured by O’Hara & Co., Ltd. (Tokyo, Japan). The lever position was monitored by a rotary encoder (MES-12-2000P), which continuously recorded results at an acquisition rate of 1000 Hz using a NI-DAQ (PCIe-6321; National Instruments, Austin, TX, United States). The sound, water, and air-puff stimulus were controlled using a program written in LabVIEW (2018, National Instruments, RRID: SCR_014325).

#### 2.3.1 Pre-training

Training was conducted once a day, with the mice in the chamber with a head plate during the training. On the first 2–3 days, a go cue (pink noise, 0.3 s) was presented and a water reward was delivered if the mouse licked a spout within 1 s of the go cue presentation. The mice gradually learned to obtain the water reward by licking the spout after the go cue. They were then moved on to the next task, in which they had to pull the lever more than 1.6 mm for longer than 0.2 s to obtain the reward, rather than just licking the spout. The weight of the lever was fixed at 0.07 N. Over 2–3 days, the mice learned to pull the lever for a duration of more than 0.2 s within 1 s after the go cue was presented. During the last 3–4 days, two tones (6 and 10 kHz pure tones, each of duration 0.8–1.2 s) were alternately presented before the go cue at an equal probability (50%). The next trial started 3–4 s after the last timepoint at which the lever was returned to the home position (after the lever went below the 1.6 mm threshold), or after the presentation of the previous tone cue when the lever did not exceed the threshold. If a mouse pulled the lever during tone presentation, it was counted as an early pull and the tone was prolonged until the mouse stopped pulling the lever and waited for 1.5–2.5 s without pulling the lever again. Pre-training was considered complete when the lever-pull rate was >90% and the early pull rate was <30% for both tones.

#### 2.3.2 Air-puff and omission tasks

In the two-tone lever pull task, either of the tone cues used in the pre-training sessions was randomly presented. The probabilities of tone A and tone B presentations were 20 and 80%, respectively, for the air-puff task, and 40 and 60%, respectively, for the omission task. The mouse heads were fixed in a way that allowed them to pull the lever within 1 s after the cue presentation, as in the pre-training sessions. In the air-puff task, when mice pulled the lever over the threshold (1.6 mm) for longer than 0.2 s, they always received a 4 μL drop in response to either tone cue, and simultaneously received an air-puff (0.3–0.4 Mpa for 20 ms) at probabilities of 90 and 10% in response to tone A and B trials, respectively. Air-puffs were delivered from a needle tip that was located 3–5 mm away from the left eye. In the omission task, mice that pulled the lever in response to tone A and tone B received a 4 μL drop of water at probabilities of 10 and 90%, respectively, but never received the air-puff punishment. Mice that did not pull the lever did not receive water or an air-puff. The next trial started 3–4 s after the last timepoint at which the lever was returned to the home position (after the lever went below the threshold), or after the presentation of the previous tone cue when the lever did not exceed the threshold (1.6 mm). In both tasks, training was considered successful when the lever-pull rates in response to tone A and tone B were < 50% and > 50%, respectively, for two consecutive sessions (successful threshold sessions) by the tenth (air-puff task) or eighth (omission task) training session. The force required to pull the lever varied from 0.05–0.07 N per mouse, but tended to be greater for the omission task. The early pull rates in the last training session were 0.0 for both tones in the air-puff task (*n* = 5), and 0.027 ± 0.002 and 0.053 ± 0.006 for trials with tone A and tone B, respectively, in the omission task (*n* = 7). Trials with early pulls were not included in the behavioral analysis and computational modeling.

In a pilot experiment conducted prior to the start of this study, when the presentation probability of tone A in the air-puff task was set at 40%, the lever-pull rate became very low in both tone A and B trials (0.15 ± 0.21 for tone A trials and 0.20 ± 0.18 for tone B trials in the 7th training session, *n* = 3 mice). This was likely due to an increased frequency of receiving the air-puff, which led to a reduction in motivation for the task itself. Therefore, we set the presentation probability of tone A at 20% in the air-puff task. By contrast, in the omission task, when the presentation probability of tone A was set at 20%, the lever-pull rate remained high in tone A trials (0.87 ± 0.15 in the 7th training session, *n* = 5 mice). We considered that this was because, regardless of whether tone A or B was presented, if the mice pulled the lever, they received a sufficient amount of reward due to the large number of tone B trials. Therefore, we set the presentation probability of tone A to 40%.

### 2.4 Pharmacological inactivation

Four mice (two in the air-puff task and two in the omission task) performed the sessions with pharmacological inactivation (ACSF and muscimol sessions) without any training session after the second successful threshold session (Supplementary Table S1). The other mice continued to perform 1–8 training sessions until the ACSF and muscimol sessions started (Supplementary Table S1). The mice underwent bilateral craniotomies over the prefrontal cortex (ML 0.2 mm, AP 1.8 mm, diameter 1 mm) at least 3 days before the day of injection and the craniotomies were covered with silicone elastomer (Kwik-Cast, World Precision Instruments, FL, United States). Before injection, a glass pipette (3–000-203-G/X, Drummond Scientific Company, PA, United States) was pulled, cut until its outer diameter was around 40 μm, and then backfilled with mineral oil (Nacalai Tesque, Kyoto, Japan). The pipette was fitted to a Nanoject III Programmable Nanoliter Injector (Drummond Scientific Company), and ACSF or muscimol (60 nL; 5 μg/μL; M1523, Sigma-Aldrich, MO, United States) diluted in ACSF was front-loaded. During the intracranial injection, the mice were anesthetized with isoflurane (0.7–1.4%) inhalation and fixed with a head plate. The pipette tip was inserted into the brain at a depth of 1.5 mm from the cortical surface, and ACSF or muscimol was injected via a Nanoject III at a rate of 8–10 nL/min. The pipette was maintained in place for 5 min after the injection and then slowly withdrawn. Injection into the second hemisphere was always completed within 15 min from the beginning of injection into the first hemisphere. The craniotomies were covered with silicone elastomer. Both the air-puff task and the omission task were started 40 min after the injection. The order of ACSF and muscimol sessions was randomized for each mouse. After the completion of both sessions, NeuroTrace CM-Dil tissue-labeling paste (N22883, Thermo Fisher Scientific, MA, United States) was injected at the same stereotaxic site to confirm the ACSF and muscimol injection site.

### 2.5 Histology

Mice were anesthetized by intraperitoneal injection of a mixture of ketamine and xylazine, and were then perfused transcardially with PBS followed by a solution of 4% paraformaldehyde (09154–85, Nacalai Tesque). The brains were removed and stored in the fixative overnight, and were kept at 4°C until being coronally sectioned (100 μm sections) with a Vibratome (VT1000S, Leica, Germany). Sections were mounted in Vectashield mounting medium containing DAPI (H1500, Vector Laboratories, CA, United States) and were imaged with a camera (RETIGA2000, *Q*IMAGING, AZ, United States) attached to a fluorescence microscope (BX53, Olympus, Tokyo, Japan).

### 2.6 Analysis of behavioral data

The data were analyzed using MATLAB (R2023b; MathWorks, Natick, MA, United States, RRID: SCR_001622). No apparent abnormal behavior was observed on the day after a break (e.g., on a Monday). Therefore, the behavior of the mice was analyzed throughout the first to the last session. To remove the possible period within each session in which mice were less motivated, the behavioral data were determined only from the first trial to the last trial prior to obtaining 60% of the total planned reward. The lever-pull rate was defined as the number of successful lever-pull trials divided by the total number of the trials except for those with early pulls. To visualize the change in pulling or not pulling within each trial, the 10-trial moving-average of the pull-choice (pull was 1 and non-pull was 0) was used.

### 2.7 Reinforcement learning models

All behavioral data were summarized as binary data with action (to pull or not), cue type, water reward, and air-puff punishment. The sequences from a single animal were concatenated through all sessions, including additional sessions after the second successful threshold session. These data series were separated into two sequences consisting of tone A and tone B trials, which were used to model the learning process of the mice.

The model was based on that used in our previous study (Tanimoto et al., 2020). The values for pulling and not pulling the lever during the t-th trial of each tone cue (*x* ∈ {A, B}) were defined as $Q_{x,\text{pull}}\left(t\right)$ and $Q_{x,\text{non}-\text{pull}}\left(t\right)$, respectively. When mice pulled the lever, $Q_{x,\text{pull}}\left(t\right)$ was updated according to Equation (1) and $Q_{x,\text{non}-\text{pull}}\left(t\right)$ was updated according to Equation (2). When mice did not pull the lever, $Q_{x,\text{pull}}\left(t\right)$ was updated according to Equation (3) and $Q_{x,\text{non}-\text{pull}}\left(t\right)$ was updated according to Equation (4).


(1) $$\begin{array}{l} Q_{x,\text{pull}}\left(t+1\right)=Q_{x,\text{pull}}\left(t\right)+\alpha_{l}\;\\ \left(\kappa_{r}*r_{x}\left(t\right)-\kappa_{p}*p_{x}\left(t\right)-Q_{x,\text{pull}}\left(t\right)\right) \end{array}$$

(2) $$Q_{x,\text{non}-\text{pull}}\left(t+1\right)=\left(1-\alpha_{f}\right)\;Q_{x,\text{non}-\text{pull}}\left(t\right)$$

(3) $$Q_{x,\text{pull}}\left(t+1\right)=\left(1-\alpha_{f}\right)\;Q_{x,\text{pull}}\left(t\right)$$

(4) $$Q_{x,\text{non}-\text{pull}}\left(t+1\right)=Q_{x,\text{non}-\text{pull}}\left(t\right)+\alpha_{l}\;\left(\psi -Q_{x,\text{non}-\text{pull}}\left(t\right)\right)$$

where *α*_l_ (0 < *α*_l_ < 1) was the learning rate, *α*_f_ (0 < *α*_f_ < 1) was the forgetting rate, *κ*_r_ (≥ 0) was the subjective goodness of a water reward (reward value), *κ*_p_ (≥ 0) was the subjective strength of aversion to air-puff punishment, and ψ (≥ 0) was the goodness of the covert reward, which is assumed to be constantly obtained as a result of a non-pull (i.e., the saving of the cost accompanying the lever-pull) (Lee et al., 2016; Cheval et al., 2018; Tanimoto et al., 2020). In both the air-puff task and the omission task, $r_{x}\left(t\right)$ corresponded to the water reward (presence, 1; absence, 0) in the *t*-th tone *x* trial. In the air-puff task, $p_{x}\left(t\right)$ was defined as 1 when mice pulled the lever and an air-puff was delivered in the *t*-th tone *x* trial, whereas $p_{x}\left(t\right)$ was 0 when mice did not pull the lever or when they pulled the lever but an air-puff was not delivered. In the omission task, $p_{x}\left(t\right)$ was defined as 1 when the mice pulled the lever but water was not delivered in the *t*-th tone *x* trial, whereas $p_{x}\left(t\right)$ was 0 when the mice did not pull the lever or when they pulled the lever and water was delivered. The pull-choice probability for the (t+1)-th trial for tone X, $P_{x,\text{pull}}\left(t+1\right)$, was calculated according to Equation (5).


(5) $$P_{x,\text{pull}}\left(t+1\right)=\frac{1}{1+\exp\left\{-\left(Q_{x,\text{pull}}\left(t\right)-Q_{x,\text{non}-\text{pull}}\left(t\right)\right)\;\right\}\;}$$

The parameters used for each model are summarized in Supplementary Table S2.

Maximum log likelihood estimation was used to fit the parameters used in all models. The likelihood (*L*) was calculated according to Equation (6):


(6) $$L=\prod_{t}z\left(t\right)$$

where z(*t*) could be calculated as $P\left(t\right)$ if the lever was pulled, and $1-P\left(t\right)$ if the lever was not pulled. The logarithm of this likelihood was multiplied by −1 to allow the use of the *fmincon* function in MATLAB with appropriate lower and upper bounds for each free parameter. For each model, the parameter fitting was repeated 5,000 times and the parameters for the fitting that showed the maximum likelihood (*L*_max_) was chosen. To compare the models, we used Akaike’s information criterion (AIC) and Bayesian information criterion (BIC). AIC and BIC are calculated according to Equation (7) and Equation (8), respectively.


(7) $$AIC=-2\log\left(L_{\text{max}}\right)+2K$$

(8) $$BIC=-2\log\left(L_{\text{max}}\right)+K\log\left(T_{n}\right)$$

where K is the number of free parameters to fit, and $T_{n}$ is the number of trials used for fitting.

To simulate the choice behavior in the model with the fitted values of the free parameters described above, the same sequences of tones across sessions were used as in the actual settings for each mouse, and the lever-pull choice (pull or non-pull) in each trial was calculated according to the pull-choice probability estimated by Equation (5). When the lever was pulled in the simulated t-th trial and was actually pulled in the *t*-th real trial, the actual values of $r_{x}$(t) and $p_{x}\left(t\right)$ were used. When the lever was pulled in the simulated t-th trial but was not actually pulled in the real *t*-th trial, $r_{x}$(t) and $p_{x}\left(t\right)$ were defined according to the determined probability (the air-puff task, 100% in both tone A and B trials for reward, 90 and 10% in tone A and B trials, respectively, for punishment; the omission task, 10 and 90% in tone A and B trials, respectively, for reward, 0% in both tone A and B trials for punishment). For the simulation of the training processes, the initial values of $Q_{x,\text{pull}}\left(1\right)$ and $Q_{x,\text{non}-\text{pull}}\left(1\right)$ were the same as those for the fitting. The simulation was repeated 1000 times and the pull choice was averaged in each trial. The goodness of the generative performance of each model was estimated by calculating the root mean squared error (RMSE) between the actual choice and the simulated choice averaged across 1,000 simulations. The RMSE was calculated according to Equation (9):


(9) $$\text{RMSE}=\sqrt{\frac{1}{N}\sum_{i=1}^{N}{\left({\text{Pred}}_{i}-{\text{Act}}_{i}\right)}^{2}}$$

where *N* is the total number of trials including both tone A and tone B trials, ${\text{Act}}_{i}$ indicates the actual lever-pull choice (1, pull; 0, non-pull) in the *i*-th trial, and ${\text{Pred}}_{i}$ indicates the simulated lever-pull choice (1, pull; 0, non-pull) in the *i*-th trial.

For visual presentation of mouse-averaged pull-choice behaviors the total number of trials for each mouse was divided into bins (10 in tone A trials and 40 in tone B trials for the air-puff task, 20 in tone A trials and 30 in tone B trials for the omission task), according to the probabilities of tone A and tone B presentations. Then, the average per bin was calculated for each mouse.

To better simulate the pull-choice behavior in the ACSF and muscimol sessions, the parameters in the S-F and the P-S-F models were modulated (parameter optimization). Search ranges for *α*_l_ and *α*_f_ were from 0 to 1 in increments of 0.01, whereas the search ranges for *κ*_r_, *κ*_p_, and *ψ* were from 0 to 20 in increments of 0.1. One parameter was modulated and the other parameters were fixed at their original fitted values. The last *Q*-values of the model fitting in the training sessions were used as the initial *Q*-values in the ACSF and muscimol sessions. The simulation for each value was repeated 1,000 times and the RMSE was calculated according to Equation (9). We considered the parameter that minimized RMSE to be the one that most reflected the effect of the injection.

In the modified version of the parameter optimization in the air-puff task, we assumed that the initial *Q*-values already reflected the value of the parameter that was newly modulated because the injection was conducted before the ACSF or muscimol session started. If Equations (1) and (4) were in a steady-state and the mice recognized the expected values of *r*_A_(*t*), *r*_B_(*t*), *p*_A_(*t*), and *p*_B_(*t*) in the air-puff task as the constant values $R_{A}$ = 1, $R_{B}$ = 1, $P_{A}$ = 0.9, and $P_{B}$ = 0.1, respectively, the initial *Q*-values should be as follows:


(10) $$Q_{x,\text{pull}}\left(1\right)=\kappa_{r}*R_{x}-\kappa_{p}*P_{x}$$

(11) $$Q_{x,\text{non}-\text{pull}}\left(1\right)=\psi$$

We included these *Q*-values in the simulation and searched for the parameter that minimized the RMSE.

### 2.8 Quantification and statistical analysis

Groups were compared using independent *t-*tests or paired *t*-tests as appropriate. All statistical tests were two-tailed.

## 3 Results

### 3.1 Both positive and negative punishments suppress the reward-seeking behavior of mice

**Figure 1:**
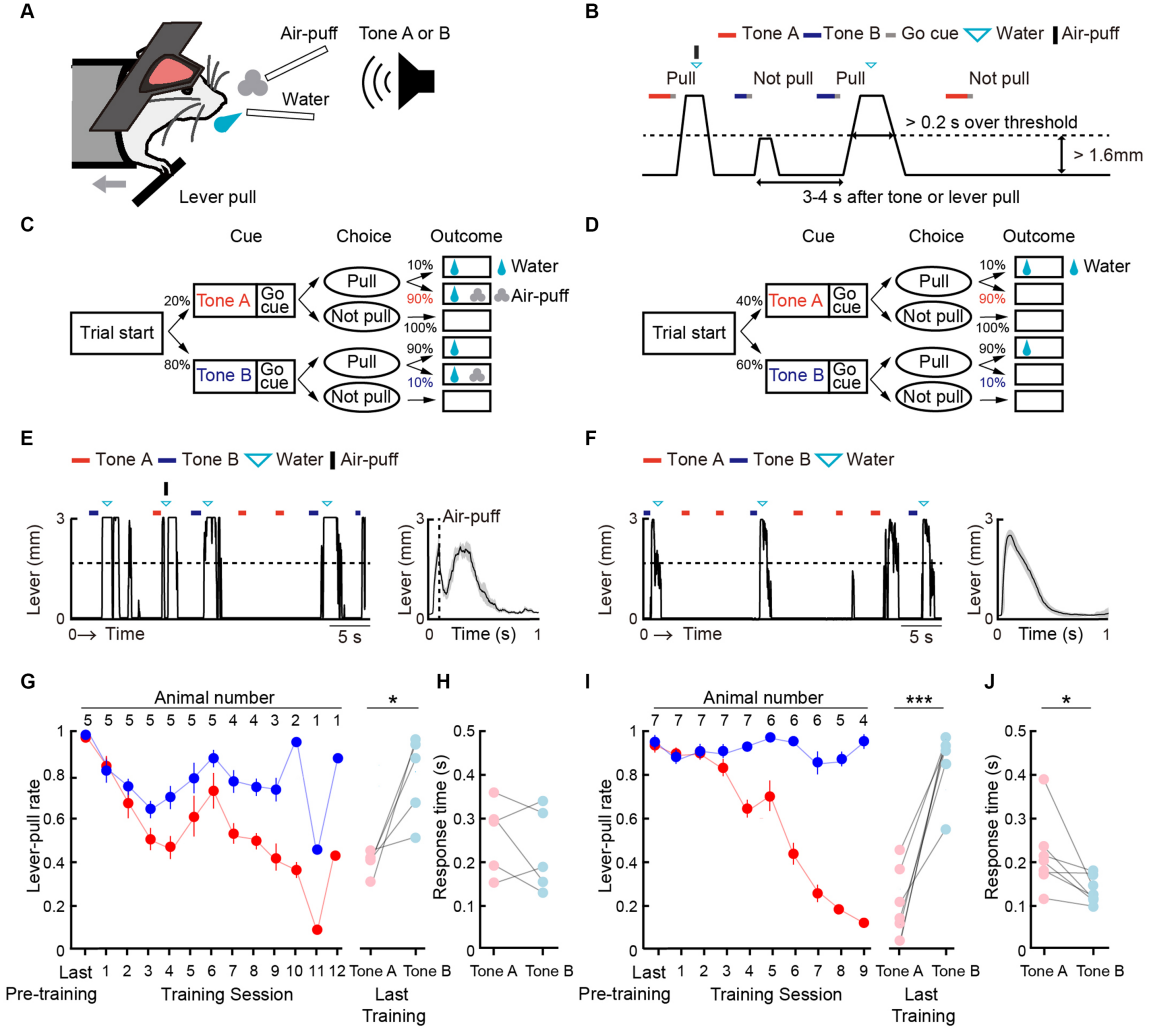
Both positive and negative punishments suppress the reward-seeking behavior of mice. **(A)** Behavioral task setup. **(B)** Schematic diagram of the air-puff task and the omission task. Black line, lever trajectory. Red horizontal bar, tone A presentation. Blue horizontal bar, tone B presentation. Gray horizontal bar, go cue presentation. Cyan inverted-triangle, water reward delivery. Black vertical bar, air-puff delivery. The air-puff was not delivered in the omission task. **(C)** Structure of the air-puff task. The air-puff was delivered at 90% of tone A trials with lever-pull and 10% of tone B trials with lever-pull. The water reward was delivered at 100% of trials with lever-pull regardless of tone type. **(D)** Structure of the omission task. The water reward was omitted at 90% of tone A trials with lever-pull and 10% of tone B trials with lever-pull. **(E)** Left, representative lever trajectories for one mouse (AM1) in the air-puff task in the last (12th) training session. Right, trial-averaged lever trajectories with air-puff in the last training session aligned to the onset of the go cue presentation. Both tone A and B trials for all mice are included. The shading indicates ± standard error of the mean (SEM) across the animals (*n* = 5). **(F)** Left, representative lever trajectories for one mouse (OM1) in the omission task in the last (8th) training session. Right, trial-averaged lever trajectories with reward omission in the last training session aligned to the onset of the go cue presentation. Both tone A and B trials for all mice are included. The shading indicates ±SEM across the animals (*n* = 7). **(G,I)** Left, averaged time course of the lever-pull rate in tone A (red) and B (blue) trials in the last pre-training session and during the training sessions in the air-puff task (*n* = 5) **(G)** and omission task (*n* = 7) **(I)**. The number above each session indicates the number of animals in each session. Right, the lever-pull rate in the last training session in tone A (red) and tone B (blue) trials. Light colored dots indicate individual mice. *p = 0.0134, ***p = 0.0008, paired *t*-test. **(H,J)** Response time in tone A (red) and B (blue) trials in the last training session of the air-puff task **(H)** and the omission task **(J)**. Light colored dots indicate individual mice. In **(H)**, the response time was not different between tone A and B trials (*p* = 0.58, *n* = 5, paired *t*-test), whereas in **(J)**, it was different (*p = 0.035, *n* = 7).

We modified a two-tone lever-pull task for head-fixed mice that we previously reported (Tanimoto et al., 2020) into a two-tone lever-pull task with the risk of two types of punishment: positive punishment (i.e., exposure to an air-puff) and negative punishment (i.e., omission of a reward) (Figures 1A,B). Two pure tones (6 and 10 kHz pure tone for 0.8–1.2 s), the reward-seeking action (pulling the lever after the go sound cue [pink noise] that followed the tone presentation), and the reward (a water drop delivered from a spout near the mouth) were common to both tasks. In the two-tone lever-pull task with the risk of an air-puff (“air-puff task”), if the head-fixed mouse pulled the lever after the go cue presentation, a water reward was delivered at a probability of 100% regardless of the tone type. In addition, an air-puff (0.3–0.4 Mpa) was delivered at a probability of 90% after the lever was pulled in each trial with a tone A presentation (tone A trial) and a probability of 10% after the lever was pulled in each trial with a tone B presentation (tone B trial). Tones A and B were randomly presented at probabilities of 20 and 80%, respectively. If the animal did not pull the lever, they did not receive the air-puff or the water reward (Figure 1C). In the two-tone lever-pull task with a risk of reward omission (“omission task”), the water reward was delivered at a probability of 10% after a lever-pull in each tone A trial and at a probability of 90% after a lever-pull in each tone B trial. This corresponded to reward omission probabilities of 90 and 10% for tone A and B trials, respectively. Tones A and B were randomly represented at probabilities of 40 and 60%, respectively. No air-puff was delivered in the omission task, and if the mice did not pull the lever, they did not receive the water reward (Figure 1D).

After pre-training with the reward delivery at 100% probability after lever-pulls in both tone A and B trials without the air-puff, the air-puff was introduced to the air-puff task and the reward omission was introduced to the omission task. In both tasks, as the training progressed, the lever-pull rate (the number of successful lever-pull trials divided by the number of total trials except for those with lever pulling before the go cue presentation) became lower in tone A trials than in tone B trials (Figures 1E,F). The air-puff disturbed the lever-pull trajectory (Figure 1E). If mice showed lever-pull rates <0.5 in tone A trials and > 0.5 in tone B trials for two consecutive training sessions (threshold sessions), we considered that they had learned the task and they were subsequently subjected to two sessions with pharmacological experiments, one with injection of artificial cerebrospinal fluid (ACSF) into the mPFC and one with injection of muscimol into the mPFC (ACSF and muscimol sessions, respectively). The mice that performed all the training, ACSF, and muscimol sessions with the air-puff (*n* = 5) and omission (*n* = 7) tasks were used for the analyses in the current study.

In these mice, the lever-pull rate in tone A trials gradually decreased in both air-puff and omission tasks (Figures 1E,G). As mice required different numbers of sessions to meet the successful training criteria, the number of animals decreased as the session progressed (Supplementary Table S1). In the aripuff task, the lever-pull rate in tone B trials slightly decreased in the first few sessions and then gradually increased (Figure 1G). On the other hand, the lever-pull rate in tone B trials in the omission task remained high (> 0.8) (Figure 1I). As a result, in the last training session, the lever-pull rates between tone A and B trials were different in both air-puff and omission tasks (Figures 1G,I). Response time (the latency between cue presentation and the onset of movement) is frequently used to estimate attention and reward expectations (Robbins, 2002), and in the last training session, this time was similar between tone A and B trials for the air-puff task (Figure 1H), but was longer in tone A trials than in tone B trials for the omission task (Figure 1J). This suggests that the response time did not simply reflect the magnitude of punishment expectation, but that it reflected the magnitude of reward expectation to some extent.

### 3.2 The air-puff is well modeled as positive punishment in the framework of reinforcement learning

To better understand how positive punishment (air-puff) and negative punishment (reward omission) affected the choice behavior (pull or non-pull) of the mouse, we constructed *Q*-learning models with a maximum of five parameters (Palminteri et al., 2015; Tanimoto et al., 2020) to predict the choice behavior during the training sessions in the air-puff and omission tasks (Supplementary Table S1; see STAR Methods for details). On the basis of our previous study (Tanimoto et al., 2020), we assumed that these tasks included two choices, pull and non-pull, and there were values of pulling the lever (*Q*_pull_) and non-pulling of the lever (*Q*_non-pull_) for both tone A and B trials in each task. The pull-choice probability in each trial was determined from the sigmoidal function of the difference between *Q*_pull_ and *Q*_non-pull_ in that trial. To update *Q*-values, we introduced the following terms: a reward value term (*κ*_r_) that increased *Q*_pull_ after the reward acquisition, a punishment term (*κ*_p_) (Lim et al., 2021) that reduced *Q*_pull_ after the punishment was delivered (air-puff stimulus in the air-puff task and reward omission in the omission task), and a saving term (*ψ*, equal to a covert reward for non-pull) that increased *Q*_non-pull_ after the lever was not pulled. We introduced the term *ψ* because we previously found that *ψ* well explained the decrease in the pull probability in tone A trials during training for a similar omission task^5^. We also added a learning rate (*α*_l_) and forgetting rate (*α*_f_), which represent the change rates of *Q*_pull_ and *Q*_non-pull_ (Barraclough et al., 2004; Ito and Doya, 2009). In all models, *κ*_r_ and *α*_l_ were included. Seven models were constructed depending on how the use of *κ*_p_, *ψ*, and *α*_f_ was combined (Supplementary Table S1). To estimate the predictive performance of the models, we used Akaike’s information criterion (AIC) and Bayesian information criterion (BIC) (Daw, 2011).

**Figure 2:**
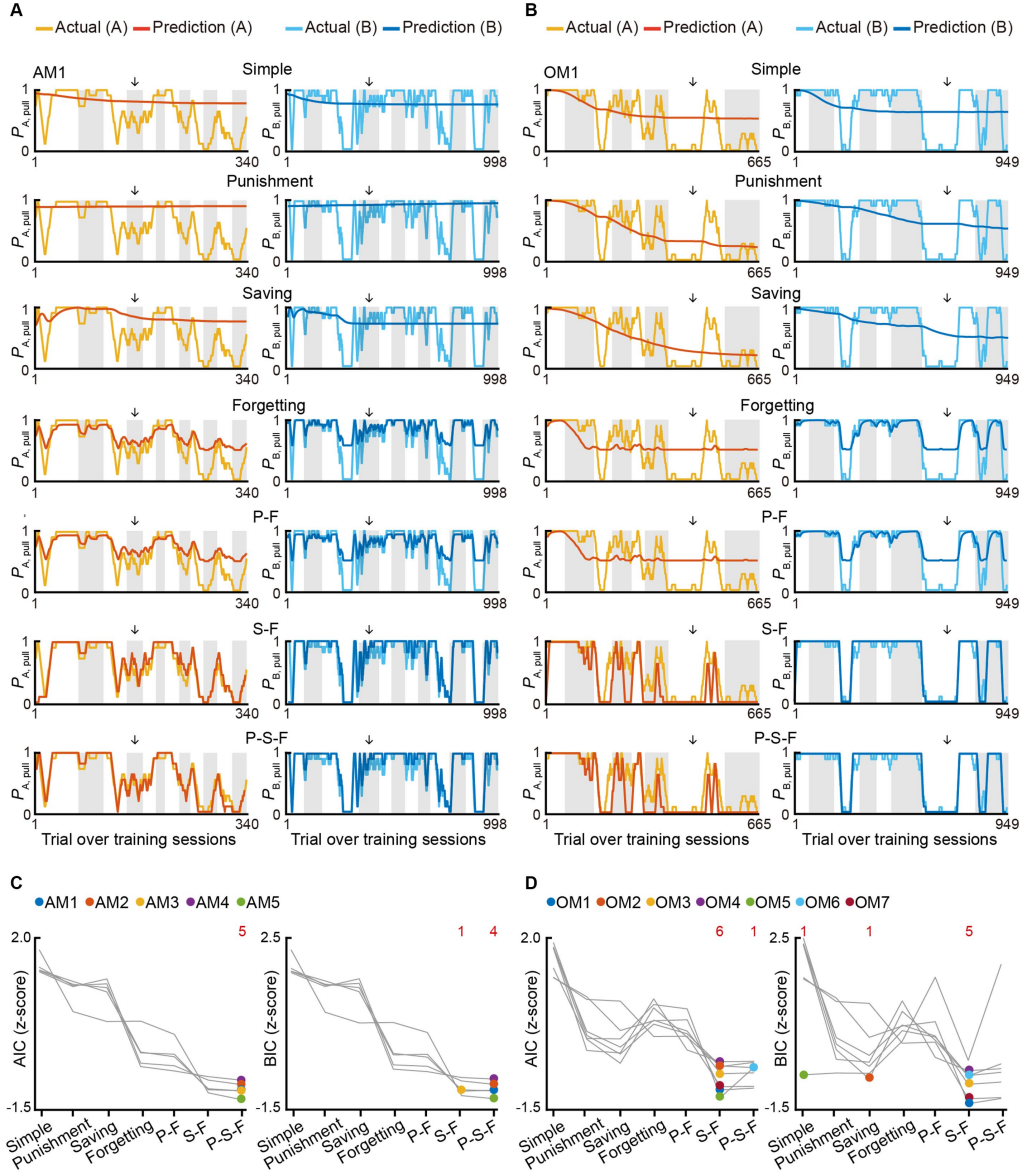
Reinforcement learning models to explain the pull-choice behavior in the air-puff and omission tasks. **(A,B)** Time course of predicted pull-choice probabilities in seven types of reinforcement learning model (simple, punishment, saving, forgetting, P-F, S-F, and P-S-F models) in tone A (left, red) and tone B (right, blue) trials for one mouse (AM1) in the air-puff task **(A)** and for one mouse (OM1) in the omission task **(B)**. The 10-trial moving averages of the actual pull choice in tone A (yellow) and tone B (cyan) trials are overlaid. Even sessions are shaded. Arrows indicate the second successful threshold session (see also Supplementary Figure S1; Supplementary Tables S1, S2). **(C,D)** Z-scored AIC (left) and BIC (right) of seven types of reinforcement learning models in the air-puff task **(C)** and omission task **(D)**. Colored dots indicate the model with the best score for each mouse. Red numbers above the graph indicate the number of dots for the corresponding model. Each color indicates a different animal (AM1–AM5 in the air-puff task and OM1–OM7 in the omission task).

In both tasks, the model with *ψ* and *α*_f_ and without *κ*_p_ (S-F model) and the model with *κ*_p_, *ψ*, and *α*_f_ (P-S-F model) appeared to explain the choice behavior with trial-to-trial variability better than the other models (Figures 2A,B; Supplementary Figures S1A–D). In the air-puff task, the P-S-F model was the best-fitting model in five and four out of five mice according to the AIC and BIC scores, respectively (Figure 2C). By contrast, in the omission task, the S-F model was the best-fitting model in six and five out of seven mice according to the respective AIC and BIC scores (Figure 2D). These results indicate that the air-puff effect was well modeled as an overt punishment, with the punishment term *κ*_p_ reducing the value of pull-choice, but the reward omission not doing this.

**Figure 3:**
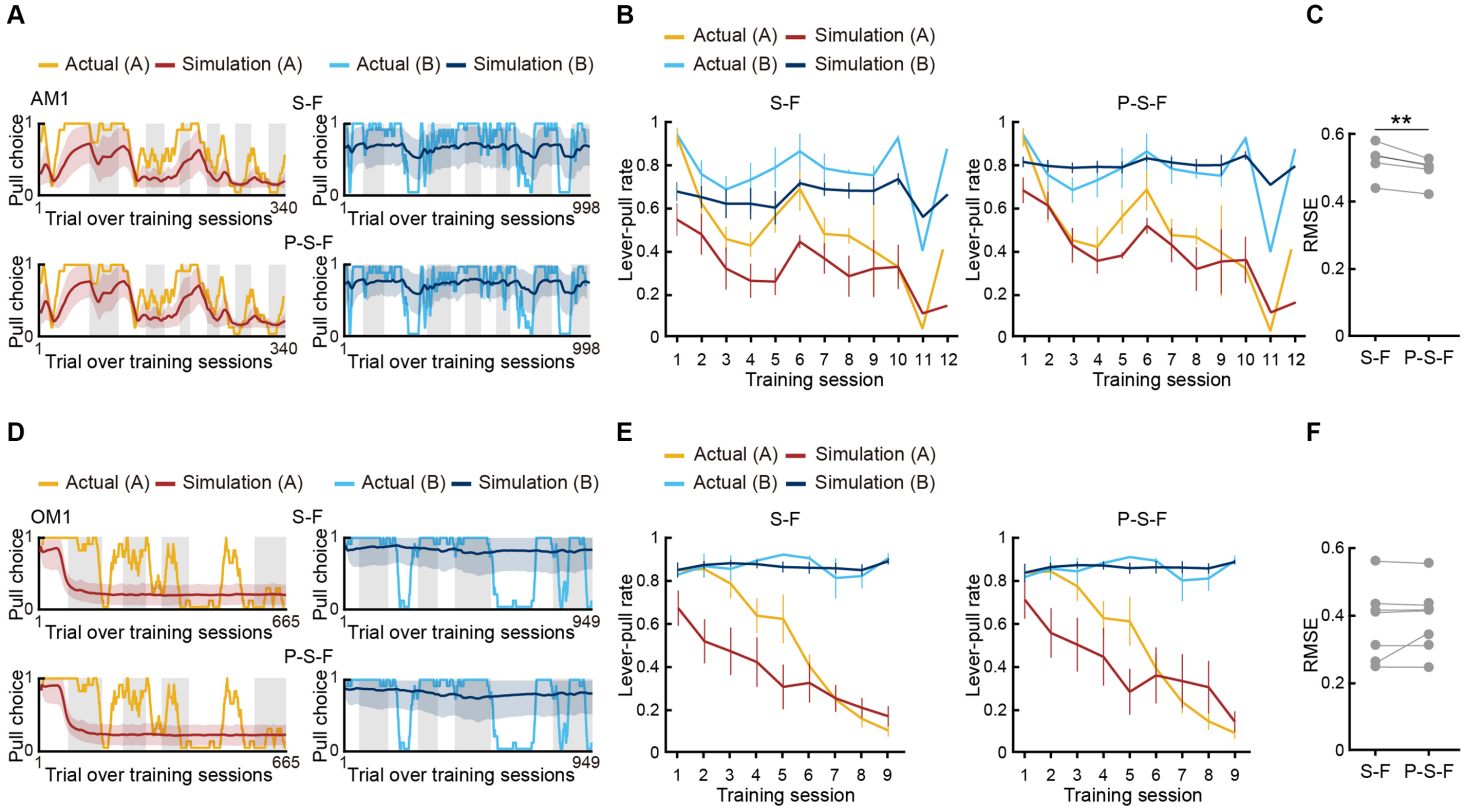
Simulation of choice behavior with S-F and P-S-F models. **(A,D)** Simulated pull-choice behaviors with S-F (upper) and P-S-F (lower) models in tone A (left) and tone B (right) trials for the mouse AM1 in the air-puff task **(A)** and for the mouse OM1 in the omission task **(D)**. For each mouse, simulation was repeated 1,000 times and the choice behavior was averaged in each trial (0–1). The 10-trial moving averages of the simulated pull choice in tone A (dark red) and tone B (dark blue) trials are shown. The shading indicates ±SD across 1,000 simulations. The yellow and cyan traces represent the 10-trial moving averages of the actual pull choices in tone A and tone B trials, respectively. Even sessions are shaded. **(B,E)** Mouse-averaged actual (tone A trials, yellow; tone B trials, cyan) and simulated (tone A trials, dark red; tone B trials, dark blue) lever-pull rates across training sessions in the air-puff task (*n* = 5) **(B)** and the omission task (*n* = 7) **(E)**. For each mouse, the simulated lever-pull rate was averaged over 1,000 simulations. The error bar indicates ±SEM across the animals. **(C,F)** The RMSE between the simulated and actual pull choices in the air-puff task **(C)** and the omission task **(F)**. Each gray line represents an individual mouse in S-F and P-S-F models. For each mouse, the RMSE was averaged over 1,000 simulations. In **(C)**, **p = 0.0083, paired *t-*test (*n* = 5). In **(F)**, *p* = 0.3950 (*n* = 7).

To examine whether the best-fitting model was also the best for generating pull-choice behavior similar to that shown by the actual mice, we conducted a model simulation (Palminteri et al., 2015, 2016; Palminteri and Pessiglione, 2017; Tanimoto et al., 2020). For each mouse, we used the fitted parameters in the S-F and P-S-F models to simulate the lever-pull choice (1, pull; 0, non-pull) in each trial in the order of the actual tone A and B trials (Figures 3A,D; Supplementary Figures S2A–D). In the air-puff task, the simulation with the P-S-F model better regenerated the time course of the actual choice behaviors in both tone A and B trials than that with the S-F model (Figure 3B). The goodness of the generative performance of each model was estimated by calculating the root mean squared error (RMSE) between the actual choice and the simulated choice. The RMSE was smaller with the P-S-F model than with the S-F model (Figure 3C). Thus, the P-S-F model was better than the S-F model as both a fitting model and a generative model. In the omission task, both S-F and P-S-F models well reproduced the choice behaviors (Figure 3E), and there was no significant difference in the RMSE of these two models (Figure 3F). Considering that there were less parameters in the S-F model (4) than in the P-S-F model (5), we concluded that the S-F model explained the choice behavior in the omission task better than the P-S-F model.

**Figure 4:**
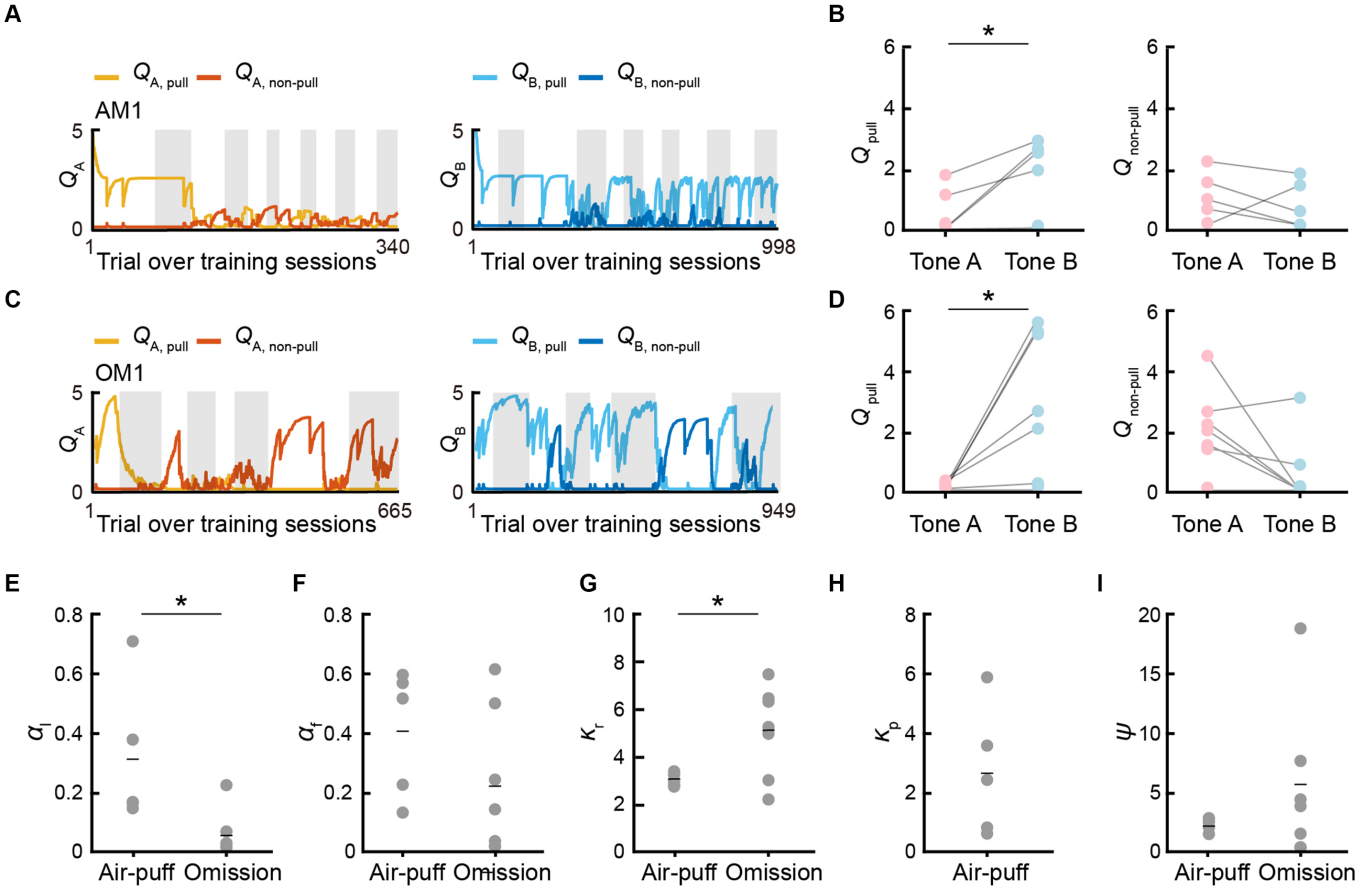
*Q*-values and model parameters in the best-fitting models. **(A,C)** Time courses of *Q*_A, pull_ (yellow) and *Q*_A, non–pull_ (red) (left), and *Q*_B, pull_ (cyan) and *Q*_B, non–pull_ (blue) (right), for the mouse AM1 in the air-puff task **(A)** and for the mouse OM1 in the omission task **(C)**. **(B,D)** Left, *Q*_A, pull_ and *Q*_B, pull_ averaged within the last training session in the air-puff task (*n* = 5) **(B)** and the omission task (*n* = 7) **(D)**. Right, *Q*_A, non–pull_ and *Q*_B, non–pull_ averaged within the last training session in the air-puff task **(B)** (*n* = 5) and the omission task **(D)** (*n* = 7). *Q*_A, pull_ vs. *Q*_B, pull_ in the air-puff task, *p = 0.0184; *Q*_A, non–pull_ vs. *Q*_B, non–pull_ in the air-puff task, *p* = 0.4780; *Q*_A, pull_ vs. *Q*_B, pull_ in the omission task, *p = 0.0195; *Q*_A, non–pull_ vs. *Q*_B, non–pull_ in the omission task, *p* = 0.0559, paired *t-*test. **(E–I)** Model parameters in the P-S-F model for the air-puff task (*n* = 5) and in the S-F model for the omission task (*n* = 7). The comparison between the air-puff and omission tasks was conducted with an independent *t-*test. **(E)** Learning rate (*α*_l_). *p = 0.0218. **(F)** Forgetting rate (*α*_f_). *p* = 0.2008. **(G)** Reward value (*κ*_r_). *p = 0.0456. **(H)** Strength of aversion to the air-puff (*κ*_p_). **(I)** Non-pull preference (*ψ*). *p* = 0.2700. Short horizontal lines indicate animal-averaged values (*n* = 5 for air-puff task, *n* = 7 for omission task).

Next, we compared *Q*_pull_ and *Q*_non-pull_ between the P-S-F model in the air-puff task and S-F model in the omission task. *Q*_pull_ in tone A trials (*Q*_A, pull_) decreased and *Q*_pull_ in tone B trials (*Q*_B, pull_) was basically high in both the air-puff and omission tasks throughout the training sessions and *Q*_B, pull_ was higher than *Q*_A, pull_ in the last session in both tasks (Figures 4A–D). By contrast, *Q*_non-pull_ in tone A trials (*Q*_A, non–pull_) and *Q*_non-pull_ in tone B trials (*Q*_B, non–pull_) in the air-puff and omission tasks fluctuated during the training sessions, and *Q*_A, non–pull_ and *Q*_B, non–pull_ was not different in the last session in both tasks (Figures 4A–D). When the parameters were compared between the two tasks (Figures 4E–I), the learning rate (*α*_l_) was higher in the air-puff task than in the omission task, the reward value (*κ*_r_) was lower in the air-puff task than in the omission task, and the saving term (*ψ*) was lower in the air-puff task than in the omission task, but the difference was not significant.

Thus, in the reinforcement learning models, there were differences between the air-puff and omission tasks, not only in respect to whether *κ*_p_ was included or not, but also in the values of *α*_l_ and *κ*_r_. The high *α*_l_ in the air-puff task might reflect the fact that the air-puff increased the attention to the task structure to avoid the positive punishment as soon as possible. The low *κ*_r_ in the air-puff task might reflect the fact that the rarity value of the reward per trial was lower in the air-puff task than in the omission task (Tanimoto et al., 2020). It is also possible that the air-puff (positive punishment) suppressed *κ*_r_ more strongly than the reward omission (negative punishment) did. The reward omission was not represented as the overt punishment *κ*_p_, but it might increase the covert reward for non-action *ψ* more than the positive punishment, and the high *ψ* would increase the non-pull choice in tone A trials. As a result, *Q*_A, non–pull_ might tend to become larger than *Q*_B, non–pull_ (Figure 4D) as previously suggested (Tanimoto et al., 2020). These results indicate that although the time course of the lever-pull rate in tone A and B trials during training sessions was relatively similar between these tasks, the learning process related to the choice behavior might be substantially different.

### 3.3 mPFC inactivation promotes lever-pull choice in the air-puff task, but not in the omission task

**Figure 5:**
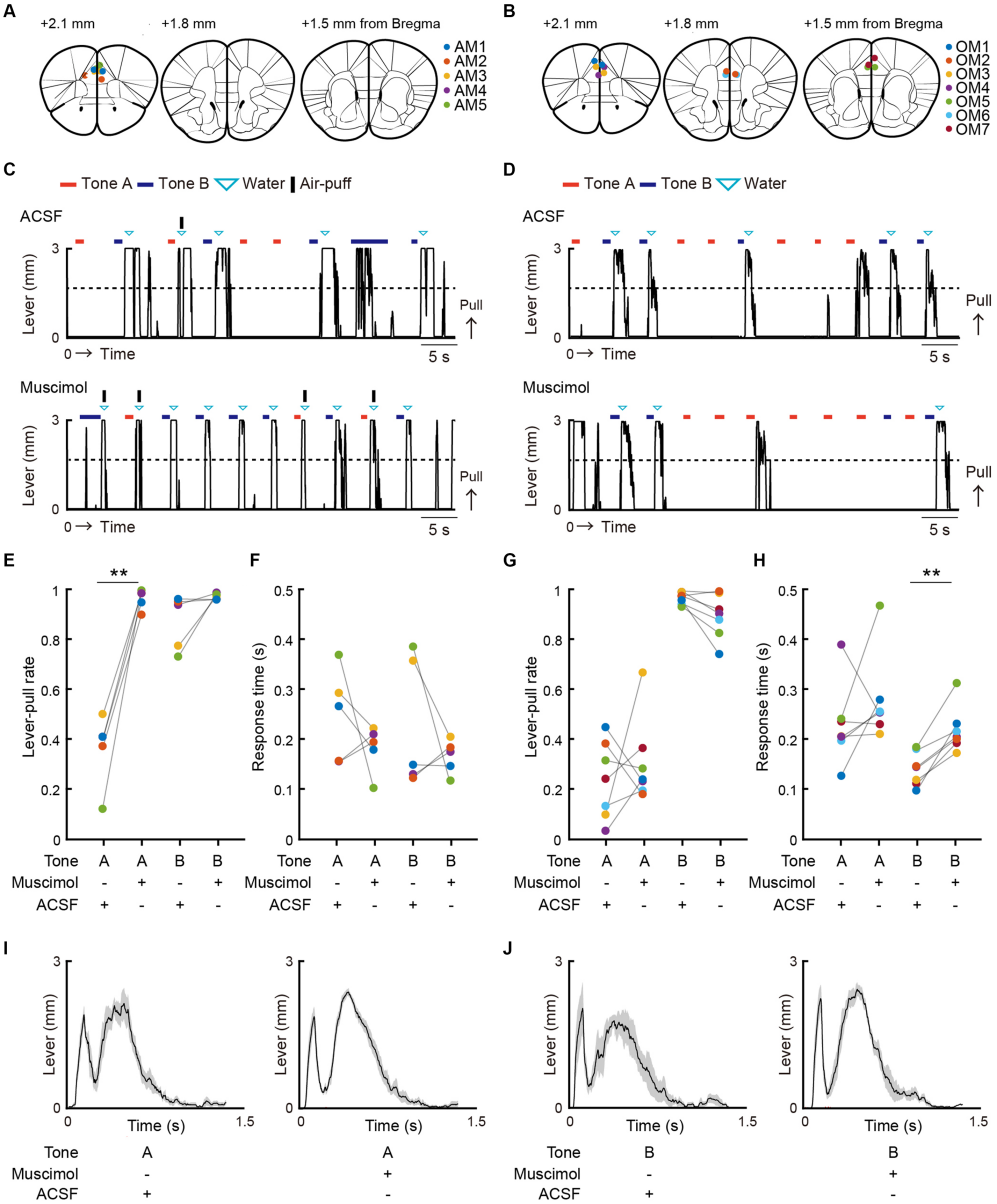
mPFC inactivation promotes lever-pull choice in the air-puff task, but not in the omission task. **(A,B)** The injection sites for ACSF and muscimol in the air-puff task **(A)** and the omission task **(B)**. The injection sites were inferred from fluorescence labeling in brain slices. Each colored dot represents an individual mouse (AM1–AM5 and OM1–OM7) (see also Supplementary Figure S3). The length from the bregma indicates the distance of the slice along the anterior–posterior axis. The atlas images are taken from *The Mouse Brain in Stereotaxic Coordinates*^48^. **(C,D)** Representative lever trajectories in the ACSF (upper) and muscimol (lower) sessions for the mouse AM1 in the air-puff task **(C)** and for the mouse OM1 in the omission task **(D)**. Black line, lever trajectory. Red bar, tone A presentation. Blue bar, tone B presentation. Cyan inverted-triangle, water reward delivery. Black horizontal bar, air-puff delivery (see also Supplementary Figures S3, S5). **(E)** Lever-pull rate in tone A (red) and B (blue) trials in the ACSF and muscimol sessions of the air-puff task (*n* = 5). Each colored line represents an individual mouse (AM1–AM5). ACSF vs. muscimol in tone A trials, **p = 0.0051 by paired *t*-test. ACSF vs. muscimol in tone B trials, *p* = 0.2233 by paired *t*-test (*n* = 5). **(F)** Response time in tone A (red) and B (blue) trials in the ACSF and muscimol sessions of the air-puff task (*n* = 5). Each colored line represents an individual mouse (AM1–AM5). ACSF vs. muscimol in tone A trials, *p* = 0.3216 by paired *t*-test. ACSF vs. muscimol in tone B trials, *p* = 0.3745 by paired *t*-test (*n* = 5). **(G)** Lever-pull rate in tone A (red) and B (blue) trials in the ACSF and muscimol sessions of the omission task (*n* = 7). Each colored line represents an individual mouse (OM1–OM7). ACSF vs. muscimol in tone A trials, *p* = 0.4917 by paired *t*-test. ACSF vs. muscimol in tone B trials, *p* = 0.1627 by paired *t*-test (*n* = 7). **(H)** Response time in tone A (red) and B (blue) trials in the ACSF and muscimol sessions of the omission task (*n* = 7). Each colored line represents an individual mouse (OM1–OM7). ACSF vs. muscimol in tone A trials, *p* = 0.2829 by paired *t-*test. ACSF vs. muscimol in tone B trials, **p = 0.0012 by paired *t*-test (*n* = 7). **(I,J)** Lever trajectories averaged across trials and aligned to the onset of the go cue presentation in tone A with the air-puff stimulus **(I)** and tone B trials with the air-puff stimulus **(J)** in the ACSF and muscimol sessions of the air-puff task. The shading indicates ±SEM across the animals (*n* = 5).

Next, we examined how the mPFC contributed to the choice behavior in the air-puff and omission tasks. After completing the training (where lever-pull rates were less than 0.5 in tone A trials and greater than 0.5 in tone B trials for two consecutive training sessions), we conducted sessions during which ACSF or muscimol was injected into the bilateral mPFC. The order of the ACSF and muscimol sessions was randomized for each mouse. The injection was mainly targeted into the prelimbic region (ML 0.2 mm, AP 1.8 mm, DV 1.5 mm; Figures 5A,B; Supplementary Figures S3A,D).

The ACSF injection did not appear to change the choice behavior in either task (Figures 5C,D). By contrast, the muscimol injection increased the pull choice in tone A trials in the air-puff task (Figures 5C,E), although the response times in both tone A and B trials did not show a large change (Figure 5F). However, the muscimol injection did not change the pull choice in tone A trials in the omission task (Figures 5D,G). Furthermore, the response time in tone A trials was similar between the muscimol and ACSF sessions, whereas in tone B trials it was longer in the muscimol session than in the ACSF session (Figure 5H).

One possible mechanism to explain the change in the lever-pull rate following muscimol injection in the air-puff task was that mPFC inactivation reduced the sensory response to the air-puff. If this were the case, we would anticipate less disturbance in lever-pull trajectory after an air-puff in muscimol sessions versus ACSF sessions. However, the lever-pull movement was equally disrupted after the air-puff with both tone types in the muscimol and ACSF sessions (Figures 5I,J). This result suggests that although the mice with mPFC inactivation sensed the air-puff in terms of the withdrawal response, they came to frequently pull the lever regardless of the probability of the air-puff stimulus.

### 3.4 mPFC inactivation increases sensitivity to reward in the air-puff task

Here, we hypothesized that in the air-puff task, the mPFC inactivation changed some internal state that was specifically related to one of the variables in the best-fitting reinforcement learning model, but that this internal state change did not happen in the omission task. In particular, we suspected that the strength of aversion to the air-puff (*κ*_p_) might be reduced in the muscimol session with the air-puff task, and therefore the value of the lever-pull increased. To test this, we investigated which model parameter best explained the change in choice behavior in the muscimol session, using approaches similar to those used in primate studies (Ahn et al., 2008; Eisenegger et al., 2014; Costa et al., 2015; Palminteri et al., 2016; Rouhani and Niv, 2019).

**Figure 6:**
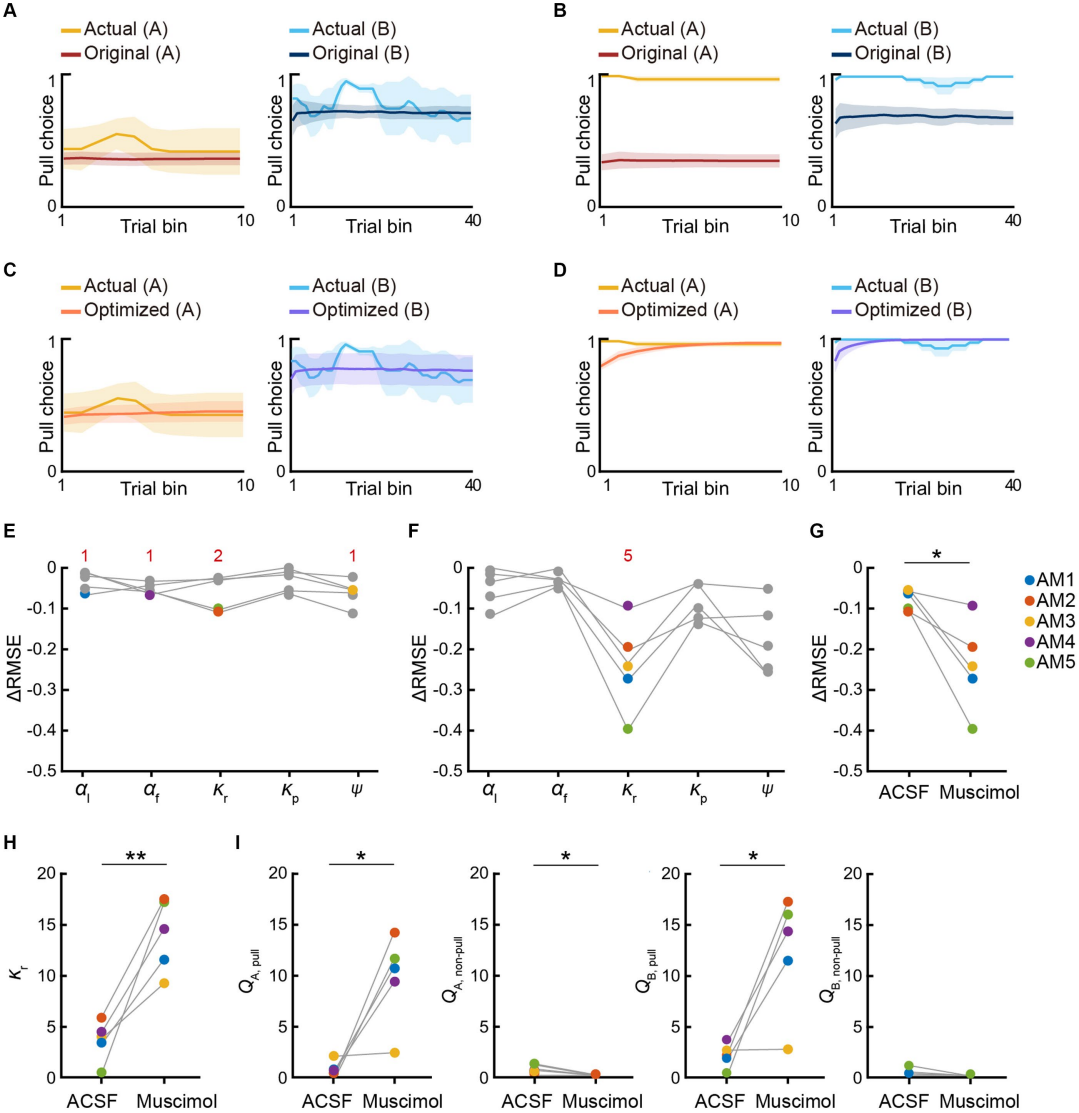
mPFC inactivation increases sensitivity to reward in the air-puff task. **(A,B)** Simulated pull-choice behaviors in the P-S-F model with the best parameter set for the training sessions in the ACSF session **(A)** and muscimol session **(B)** in the air-puff task. For each mouse, the simulation was repeated 1,000 times and the choice behavior was averaged in each trial (0–1). To average the choice behavior across the mice with different trial numbers, the trials in the session were divided into 10 bins in tone A trials and 40 bins in tone B trials for each mouse, and the choice behavior was averaged within each bin. Finally, for each bin, the choice behavior was averaged over the mice. The yellow and cyan traces represent the actual pull choices, whereas the dark red and dark blue traces represent the pull-choice simulated with the original parameters. The shading indicates ±SEM across the animals (*n* = 5). **(C,D)** Simulated pull-choice behaviors in the P-S-F model after the parameter optimization in the ACSF session **(C)** and muscimol session **(D)** in the air-puff task. The yellow and cyan traces represent the actual pull choices, whereas the orange and purple traces represent the pull-choice simulated with the parameters after parameter optimization. The shading indicates ±SEM across the animals (*n* = 5). **(E,F)** Improvement of the simulation of the choice behavior after the parameter optimization of each parameter in the ACSF session **(E)** and muscimol session **(F)** in the air-puff task. Each line indicates a different animal (AM1–AM5). Each colored dot indicates the parameter that minimized the error between the actual and simulated choice behaviors (ΔRMSE) for each mouse. Red numbers indicate the number of the colored dots in the corresponding parameter. **(G)** The change in the RMSE (ΔRMSE) resulting from parameter optimization in the simulation of the behaviors in the ACSF and muscimol sessions. Each colored line indicates an individual mouse. *p = 0.0237, paired *t*-test. **(H)** *κ*_r_ after the parameter optimization in the ACSF and muscimol sessions. Each color indicates a different animal (AM1–AM5). **p = 0.0052, paired *t*-test. **(I)** *Q*_A, pull_, *Q*_A, non–pull_, *Q*_B, pull_, and *Q*_B, non–pull_ estimated with the parameters obtained after parameter optimization in the ACSF and muscimol sessions. Each line represents an individual mouse. Each dot indicates the average within each session. *Q*_A, pull_, *p = 0.0185; *Q*_A, non–pull_, *p = 0.0304; *Q*_B, pull_, *p = 0.0214; *Q*_B, non–pull_, *p* = 0.1291, paired *t*-test (*n* = 5). Each colored line indicates an individual mouse (AM1–AM5).

First, we used the parameter set that best explained the choice behavior during the training sessions to simulate the choice behavior in the subsequent ACSF and muscimol sessions, using the P-S-F and S-F models to simulate the choice behavior in the air-puff and omission tasks, respectively. Second, to identify the parameter that best explained the change in the choice behavior in response to the muscimol injection, the value of only one of the parameters (*α*_l_, *α*_f_, *κ*_r_, *κ*_p_, and *ψ*) was changed at a time to minimize the difference between the actual and simulated choice behaviors (Figures 6A–F). The parameter that minimized the RMSE between the actual and simulated choice behavior was considered to be the injection-related parameter (Figures 6E,F). We refer to this second calculation process as parameter optimization.

In the air-puff task, the parameter set that best explained the behaviors during the training sessions well simulated the choice behavior in the ACSF session (Figure 6A; Supplementary Figure S3B), and the parameter optimization did not greatly improve the accuracy of the simulation (Figures 6C,E; Supplementary Figure S3B). The injection-related parameters were different across the five animals (Figure 6E). These results indicate that the behavior in the ACSF session largely inherited the internal states present during the training session. By contrast, the parameter set for the training sessions did not well simulate the behaviors in the muscimol session (Figure 6B; Supplementary Figure S3C). However, after the parameter optimization, the increased lever-pull choice in tone A trials was well simulated (Figure 6D; Supplementary Figure S3C). In four of five mice, the goodness of the simulation was substantially improved (Figure 6F). The change in the RMSE (ΔRMSE) resulting from the parameter optimization was more pronounced in the muscimol session than in the ACSF session (Figure 6G). In this optimization, the injection-related parameter was the same across all five mice: the reward value *κ*_r_ increased substantially in all mice (Figure 6H). As a result, the action values (*Q*_pull_) in both tone A and B trials increased (Figure 6I).

The difference between the actual and simulated choice behaviors with the parameter set for the training sessions was particularly apparent in the early part of the muscimol session (Figures 6B,D; Supplementary Figure S3C). Thus, the increase in *κ*_r_ might just be sufficient to minimize this difference as rapidly as possible. To test this hypothesis, we also considered the potential effects of muscimol injection on the initial *Q*-values. Assuming that these values reached a steady-state value after the training sessions, we calculated them from the parameter set (see Methods for details). In this case, the decrease in *κ*_p_ and the decrease in *ψ*, as well as the increase in *κ*_r_, should increase the action value and pull-choice probability, even in the first tone A trial. However, even if we included the initial action value in the parameter optimization, *κ*_r_ was chosen as the injection-related parameter in all five mice (Supplementary Figures S4A–C). Regardless of whether or not the initial action values were considered in the parameter optimization, *κ*_r_, *Q*_A,pull_, and *Q*_B,pull_ were significantly larger in the muscimol session than in the ACSF session, whereas *Q*_A,non-pull_ was significantly smaller in the muscimol session (Figures 6H,I; Supplementary Figures S4D,E). In summary, for the air-puff task, the parameters that best simulated the choice behavior in the ACSF sessions varied across the mice, whereas the simulation of the choice behavior in the muscimol session was most improved by increasing the reward value (*κ*_r_) for all mice. The model therefore suggests that the increase in *κ*_r_ increased the action value (*Q*_pull_), so that the pull-choice probability was high from the start, even though the probability of the air-puff stimulus was as constantly high in tone A trials as in the training sessions.

In comparison, the injection-related parameters for the muscimol and ACSF sessions in the omission task varied across the mice (Supplementary Figures S3D–F, S5A–F). ΔRMSE was also small after the parameter optimization (Supplementary Figures S5E–G). This suggests that the internal state related to the S-F model parameters in the omission task was not changed by mPFC inactivation. This is consistent with the result that mPFC inactivation did not affect the choice behavior in the omission task.

## 4 Discussion

In the present study, we examined how the reward-seeking behavior of mice differed under the risks of positive (air-puff stimulus) and negative (reward omission) punishment. Although both punishments reduced the lever-pull rate in trials with higher risk, the underlying coding mechanisms appeared to differ within the framework of reinforcement learning. The air-puff was well modeled as the overt punishment in the framework of reinforcement learning, from both predictive and generative aspects. In addition, inactivation of mPFC increased the pull action in the trials with a higher probability of the air-puff stimulus. On the other hand, reward omission was well-explained as a reduction of reward value rather than the distinct punishment, and the mPFC inactivation had no effect on the low pull-choice in response to the tone with a high probability of reward omission (equal to a low probability of reward delivery). These results provide evidence that positive and negative punishments are encoded differently in our brains, involving distinct brain region.

In our previous study using the omission task (Tanimoto et al., 2020), the presentation probability of the tone with a higher reward probability (corresponding to tone B trials in the current study) was set to 30%, which was lower than in the current study (60%). Despite this difference, the time course of the lever-pull rate was similar in these studies and the values of model parameters were also comparable. Thus, at least in the 30–60% range of presentation probability for tone B, the strategy for choice behavior did not seem to differ significantly. Furthermore, because of the lower presentation probability of tone A in the current study compared to the previous study (40% vs. 70%), if there was a potential trigger for a strategy that ignored the low reward probability in tone A trials (e.g., a further reduction in the presentation probability of tone A), the lever-pull rate in tone A trials would more easily approach the lever-pull rate in tone B trials in the current study than in the previous one. However, in muscimol sessions of the omission task, no mouse increased the lever-pull rate in tone A trials to more than 0.8, which was within the range of the lever-pull rate in tone B trials (Figure 5G). Thus, we consider that it is unlikely that the higher probability of tone B presentation in the omission task prevented the detection of the mPFC contribution to negative punishment.

Furthermore, we applied an analytical method to adjust a reinforcement learning parameter to explain the change in choice behavior under the acute areal inactivation. As a result, we found that muscimol inactivation of mPFC in the air-puff task increased the sensitivity to reward by enhancing the subjective goodness for reward (*κ*_r_), rather than diminishing the subjective strength of aversion for air-puff stimuli (*κ*_p_). This approach holds promise for uncovering parameters represented by specific brain regions or cell types across various decision-making tasks.

### 4.1 The distinction between positive and negative punishments

Punishment is typically defined as the suppressive effects of undesirable outcomes on behaviors (Jean-Richard-Dit-Bressel et al., 2018). Various aversive events as well as the reduction or removal of a reward are known to serve as a punisher. However, in our previous study, we found that in the framework of reinforcement learning, reward omission is better understood as a positive reinforce for “doing nothing” rather than a punisher for “doing” (Tanimoto et al., 2020). In our current study, we have further confirmed it. By contrast, the air-puff stimulus was clearly identified as a punisher for “doing.” Thus, in contrast to positive punishment (air-puff), negative punishment (reward omission) may not be a punisher for undesirable behaviors, but rather a reinforcer for not engaging in undesirable behaviors. Considering the varied effects of mPFC inhibition on behavioral choice under the risk of positive and negative punishment, we suspect that the neural coding mechanisms for these two types of punishment may be distinct.

### 4.2 Necessity of mPFC for decision-making under the risk of positive punishment

The necessity of the mPFC for decision-making under risk of positive punishment is well established (Bechara et al., 2002; Kim et al., 2009; Xue et al., 2009; Chen et al., 2013; Friedman et al., 2015; Monosov, 2017; Siciliano et al., 2019; Fernandez-Leon et al., 2021; Bloem et al., 2022; Jacobs et al., 2022). For example, Friedman et al. (2015) showed that inhibition of neurons in the prelimbic region increases the probability of rats choosing a high-risk option (rats were exposed to brighter light but at the same time they could get sweeter milk chocolate) only when there are cost–benefit conflicts, and prelimbic inhibition does not affect the behavior when there is no conflict. Our result for the behavioral change in the muscimol session with the air-puff is consistent with these previous studies. In contrast to the air-puff task, mPFC inactivation did not change the choice behavior in the omission task. This result is consistent with another study that found that mPFC inactivation did not change the choice between a certain small reward and a risky large reward (St. Onge and Floresco, 2010). These results suggest that similar mPFC activity occurs in decision-making tasks of whether or not to act, as well as in many tasks involving the choosing of one action from multiple actions.

Our study on acute mPFC inactivation clearly suggests that the increased sensitivity to reward value, but not the decreased aversion strength to punishment, may be the most relevant to the mPFC-impaired behavior. This result is consistent with observations in patients with vmPFC lesions, who show hypersensitivity to reward (Bechara et al., 2002). However, since we changed only one parameter value, we do not exclude the possibility that parameters other than the reward value would also be affected. Alternatively, mPFC inactivation might change the animal’s behavioral strategy from the decision making-based behavior to a habitual behavior that cannot be explained by the reinforcement learning framework. However, in the omission task, the pull choice in tone A trials remained probabilistic, as in the other sessions, and the reaction time in tone B trials was rather lengthened (Figure 1). Thus, the behavioral change in the muscimol session of the air-puff task did not simply reflect the habitual behavior; the behavioral change needed the positive punishment. To directly test whether the increase in the pull choice was addictive and habitual, one could examine whether the lever-pull choice continues or not after the lever-pull is devaluated in the middle of the muscimol session.

The rodent mPFC consists of prelimbic and infralimbic regions and the ACC. These regions have distinct functions in reward-seeking behaviors under the risk of punishment (Xue et al., 2009; Friedman et al., 2015; van Holstein and Floresco, 2020). Considering that dmPFC and vmPFC in humans signal risk and outcome values, respectively (Xue et al., 2009), the behavioral change by the inactivation of mPFC in the air-puff task might be caused by the inactivation of both dorsal and ventral parts of mPFC, although the injection site was targeted to the prelimbic region. It is necessary to clarify how each region is related to the choice behavior and reward sensitivity under risk of positive punishment.

### 4.3 Possible neural mechanisms by which mPFC regulates the sensitivity to reward

mPFC and the ventral tegmental area (VTA) are strongly related to positive punishment on different timescales (Park and Moghaddam, 2017). VTA dopaminergic neurons display phasic excitatory responses tightly linked to each task event (cue, action, punishment, and reward), and might be involved in the short-timescale real-time neural processing of punishment to promote rapid behavioral adaptation. By contrast, mPFC neurons show the punishment-related responses that are longer than the transient activity of VTA dopaminergic neurons, and baseline activity of many of mPFC neurons are modulated (Park and Moghaddam, 2017). mPFC neurons that project to the dorsomedial striatum maintained the action value over a long timescale in a dynamic foraging task (Bari et al., 2019). Thus, mPFC might be responsible for longer-lasting impacts of punishment on motivational and emotional states. Such longer-lasting effects may consistently affect actions over the session, regardless of the presentation of punishment. In this case, the inactivation of mPFC may be better represented in the model as an increase in *κ*_r_ rather than as a decrease in *κ*_p_ (Figure 6; Supplementary Figure S4). From the initial tone A trial in the muscimol session of the air-puff task, the pull was consistently chosen. These results suggest that mPFC maintained the subjective value of the reward within an appropriate range, rather than the aversion strength to the punishment. This might result in the lower *κ*_r_ observed in the air-puff task compared with the omission task (Figure 2G). Alternatively, the low *κ*_r_ in the air-puff task might simply reflect the rarity value of the reward per trial (Tanimoto et al., 2020). To test whether mPFC encodes the subjective value of the reward, it is necessary to measure the activity of mPFC in the air-puff task (Kondo et al., 2017; Kondo and Matsuzaki, 2021) and clarify how its activity and the estimated reward value change when the reward delivery probabilities in tone A and B trials vary.

The activity of the mPFC under risk of positive punishment should be transmitted to other areas. Considering that modulation of the connections from the mPFC to the basolateral amygdala or VTA has a weak effect on reward-seeking behavior (Kim et al., 2009; Friedman et al., 2015), its major downstream site would be the striatum, which is critical for updating the action value (Kim et al., 2009). Inhibition of glutamatergic projections from the mPFC to the nucleus accumbens core and shell in the rat causes it to choose an option with high benefits-high costs, rather than a choice with low benefits-low costs (Friedman et al., 2015). The mPFC may suppress the activity of striatal medium spiny neurons by activating striatal interneurons. Medium spiny neurons in the dorsal striatum are largely suppressed under the cost–benefit conflict, with the activity peaks of striatal interneurons preceding the inhibition of peaks of medium spiny neurons (Friedman et al., 2015). Striatal medium spiny neuronsrepresent reward signals and promote reward-seeking behavior (Cox and Witten, 2019). Therefore, activation of striatal interneurons by the striatum-projecting mPFC neurons may subsequently suppress the striatal medium spiny neurons and prevent the sensitivity to reward from jumping up. If the VTA signal represents the aversion strength to the punishment (*κ*_p_), the signals from mPFC and VTA (*κ*_r_ and *κ*_p_, respectively) may be incorporated when the action value is updated in the striatum. Striatal neurons expressing dopamine D2 receptors are well known to mediate negative feedback through both positive and punishment (Danjo et al., 2014; Mitchell et al., 2014; Zalocusky et al., 2016; Blomeley et al., 2018). After learning of the risk of negative punishment, these striatal neurons may determine whether to act or not without using the signal from mPFC. However, in the omission task in the present study, it is unknown how the striatal neurons expressing D1 and D2 receptors are related to the choice behavior. To clarify this, optogenetic manipulation and measurement of the striatal neurons and dopaminergic neurons is necessary.

### 4.4 Limitations

This study has several limitations. In the current study, we head-fixed mice using a method developed in a previous study (Tanimoto et al., 2020) so that one-photon and two-photon calcium imaging could be used to detect the relevant cortical activity in future experiments (Kondo et al., 2017; Kondo and Matsuzaki, 2021; Terada et al., 2022). However, this head-fixed condition was more stressful for the mice than a free-moving condition. Therefore, it will be necessary to validate our current findings under less stressful conditions. Additionally, we fixed the combination of the reward and air-puff delivery probabilities within and across sessions. It was reported that mPFC inactivation changes choice behavior when the reward omission probability changes within a session (St. Onge and Floresco, 2010; van Holstein and Floresco, 2020; Piantadosi et al., 2021). Thus, mPFC would be required for behavioral flexibility under risk of negative punishment. It is necessary to verify this by varying the reward probability within the session in the current omission task. The probabilities of the tone A and B presentation were also fixed, and were different between the air-puff task and the omission task. These fixed conditions should create some bias in the choice behavior. In addition, since mPFC has various functions for each sub-region and projection, further examination with a more specific method for modulation, such as chemogenetics and optogenetics, is required.

## Data availability statement

The dataset and codes for replicating the fitting, prediction, and simulation analyses of the reinforcement model are available at https://github.com/monami-nishio/PFC-punishment.

## Ethics statement

The animal study was approved by the Institutional Animal Care and Use Committee of the University of Tokyo. The study was conducted in accordance with the local legislation and institutional requirements.

## Author contributions

MN: Conceptualization, Data curation, Formal analysis, Investigation, Methodology, Validation, Visualization, Writing – original draft, Writing – review & editing. MK: Conceptualization, Data curation, Investigation, Methodology, Supervision, Validation, Writing – original draft, Writing – review & editing. EY: Methodology, Supervision, Writing – review & editing. MM: Funding acquisition, Project administration, Resources, Supervision, Writing – review & editing, Conceptualization.
