# Supplementary material for: Medial prefrontal cortex suppresses reward-seeking behavior with risk of punishment by reducing sensitivity to reward
Source: Front Neurosci. 2024 Jun 5;18:1412509. doi: 10.3389/fnins.2024.1412509 (PMC11188571; doi:10.3389/fnins.2024.1412509)
Supplement: Supplementary file 2 [file Data_Sheet_2.docx]

Supplementary Material

**Table S1. The number of training sessions for individual mice**

The number of sessions before the second successful threshold session and the number of the additional sessions before the pharmacological session started.

|  | **The number of sessions to reach the second successful threshold session** | **The number of additional sessions** |
| --- | --- | --- |
| AM1 | 4 | 8 |
| AM2 | 5 | 3 |
| AM3 | 9 | 0 |
| AM4 | 10 | 0 |
| AM5 | 4 | 3 |
| OM1 | 7 | 1 |
| OM2 | 8 | 1 |
| OM3 | 6 | 3 |
| OM4 | 7 | 2 |
| OM5 | 4 | 0 |
| OM6 | 6 | 0 |
| OM7 | 8 | 1 |

|  | ***α*_l_** | ***α*_f_** | ***κ*_r_** | ***κ*_p_** | ***ψ*** |
| --- | --- | --- | --- | --- | --- |
| Simple | var. | 0 | var. | 0 | 0 |
| Punishment | var. | 0 | var. | var. | 0 |
| Saving | var. | 0 | var. | 0 | var. |
| Forgetting | var. | var. | var. | 0 | 0 |
| P-F | var. | var. | var. | var. | 0 |
| S-F | var. | var. | var. | 0 | var. |
| P-S-F | var. | var. | var. | var. | var. |

**Table S2. Summary of the free parameters used in each *Q*-learning model, related to Figure 2.**

Var.: variable.
